# Glutaraldehyde-Polymerized Hemoglobin: In Search of Improved Performance as Oxygen Carrier in Hemorrhage Models

**DOI:** 10.1155/2020/1096573

**Published:** 2020-09-01

**Authors:** Anca D. Farcas, Vlad Al Toma, Ioana Roman, Bogdan Sevastre, Florina Scurtu, Radu Silaghi-Dumitrescu

**Affiliations:** ^1^Institute of Biological Research Cluj, Cluj-Napoca 400113, Branch of NIRDBS Bucharest, Romania; ^2^Department of Chemistry and Chemical Engineering, Babeș-Bolyai University, Cluj-Napoca 400028, Romania; ^3^National Institute for Research and Development of Isotopic and Molecular Technologies, 400293 Cluj-Napoca, Romania; ^4^Department of Pathophysiology, University of Agricultural Sciences and Veterinary Medicine, Cluj-Napoca 400372, Romania

## Abstract

Hemoglobin- (Hb-) based oxygen carriers (HBOC) have for several decades been explored for treatment of hemorrhage. In our previous top-up tests, HBOC with lower *in vitro* prooxidant reactivity (incorporating a peroxidase or serum albumin to this end) showed a measurable but small improvement of oxidative stress-related parameters. Here, such HBOCs are tested in a hemorrhage set-up; ovine hemoglobin is also tested for the first time in such a setting, based on *in vitro* data showing its improved performance versus bovine Hb against oxidative and nitrosative stress agents. Indeed, ovine Hb performs better than bovine Hb in terms of survival rates, arterial tension, immunology, and histology. On the other hand, unlike in the top-up models, where the nonheme peroxidase rubrerythrin as well as bovine serum albumin copolymerized with Hb were shown to improve the performance of HBOC, in the present hemorrhage models rubrerythrin fails dramatically as HBOC ingredient (with a distinct immunological reaction), whereas serum albumin appears not feasible if its source is a different species (i.e., bovine serum albumin fares distinctly worse than rat serum albumin, in HBOC transfusions in rats). An effect of the matrix in which the HBOCs are dissolved (PBS versus gelofusine versus plasma) is noted.

## 1. Introduction

Hemoglobin-based oxygen carriers (HBOCs) have for several decades been proposed and tested for treatment of hemorrhage. For preparation of these, polymerization with glutaraldehyde has been one of the most often employed techniques; in fact, bovine hemoglobin polymerized with glutaraldehyde is currently approved for limited human use in two countries, while no other HBOC is to our knowledge currently approved for clinical use anywhere else [1–12]. Attempts to improve over the hemoglobin-glutaraldehyde polymer have been extensively reported, including copolymerization with antioxidant components (albumin, heme and nonheme peroxidases, superoxide dismutases, and small antioxidants) or with other proteins meant to interact with blood components (fibrinogen and carbonic anhydrase), encapsulation, and modulation of the matrix/solution in which the HBOC is dissolved [1, 2, 9, 10, 12–21]. Other approaches, not involving glutaraldehyde (or involving it as an auxiliary), have involved derivatization with inert high-molecular weight organic polymers (e.g., polyethylene glycol), polymerization with other bifunctional reagents, replacement of bovine hemoglobin with other hemoglobins (human, or from other organisms including plants) or even with other proteins (hemerythrin) or even protein-free approaches (oxygen-encapsulating emulsions, fluorinated hydrocarbons, and heme-based dendrimers) [8, 20, 22–24].

We have previously reported an evaluation of a range of physiological parameters in rats injected with small amounts of blood substitute candidates based on hemoglobin [25]. These candidates were mostly nanosized polycondensates/polymers, as shown by chromatographic and spectroscopic measurements [7, 13, 26–29]. Generally, they displayed distinctly varying prooxidative reactivity both *in vitro* and *in vivo* in top-up rat models where the respective HBOCs were injected in relatively small amounts in the absence of hemorrhage. Our hypothesis was that less prooxidant Hb derivatives/polymers will display less negative pathophysiological effects. All candidates appeared to behave very similarly to each other in terms of biochemical and immunological parameters, although the less redox-reactive ones were found to lead to less excessive accumulation of iron in organs and lower amounts of excess paramagnetic species in blood. Specifically, the two best performers were a copolymer with Hb with the recombinant nonheme iron peroxidase rubrerythrin (Rbr, originally from *Desulfovibrio vulgaris* [27]), and a copolymer of Hb with bovine serum albumin (BSA). Importantly, no pathological variations were noted in the biochemical and physiological parameters, including immunological ones, monitored for these two HBOCs, despite their use of proteins from widely different organisms [25]. Here, data on the effect of such blood substitute candidates in hemorrhagic Wistar rats is reported with emphasis on arterial tension (not previously reported for this set of HBOCs), survival rates (unexpectedly small compared to the 100% rate in previous top-up models), and, linked to these, strong immunological responses. Ovine hemoglobin is added and presented as an alternative Hb source, given its promising recent performance in *in vitro* tests [30]; to our knowledge, this is the first *in vivo* report of an ovine-based HBOC. Also, two alternative matrices for dissolving the HBOC (plasma and gelofusine) are explored as alternative to the previously employed PBS.

## 2. Materials and Methods

### 2.1. Reagents and Instrumentation

Standard reagents were of the same sources and stocks as previously described for the top-up HBOC experiments [25]. Hemoglobin derivatives (ovine and bovine glutaraldehyde-polymerized hemoglobin [27, 31], hemoglobin-BSA copolymer [7, 14], and hemoglobin-rubrerythrin copolymer [27]) were prepared as previously described. Rat hemoglobin was from Sigma Aldrich. For the Hb-BSA copolymer, a fraction separated using FPLC (procedure as described previously [14]) with a molecular weight higher than the monomer was separately prepared. Gelofusine (Braun Melsungen AG) was used without dilutions as a matrix for HBOCs and isoosmotic conditions were maintained. Human standardized plasma was obtained from the Department of Blood Transfusion of *I. Chiricuta* Institute of Oncology and also it was used as a matrix for HBOCs. Phosphate buffer saline was the third matrix of our tested HBOCs and was prepared according to canonical protocol in order to obtain a final pH = 7.4.

### 2.2. Experimental Animals

Healthy adult male Wistar rats weighing 160 ± 20 g, age 24 weeks, F1 generation, were provided with free access to standard rat chow and water. Rats were housed 8/cage and maintained in a light/temperature controlled room with a light/dark cycle 12/12 h under 22°C constant temperatures. The experiments and animals welfare were conducted following the guidelines of the directive 2010/63/UE.

### 2.3. Study Design

The experiment was performed on male Wistar rats randomly divided into groups of 6–10 animals each. The groups were defined as follows: C (control, not subjected to hemorrhage), H (control hemorrhage: subjected to hemorrhage but not to transfusion), Pls (subjected to hemorrhage then treated with plasma), Hb(b)/Pls (hemorrhage, transfused with glutaraldehyde-polymerized bovine hemoglobin in plasma), Hb(b)/PBS (hemorrhage, transfused with glutaraldehyde-polymerized bovine hemoglobin in PBS), Hb(ov)/Pls (hemorrhage, transfused with glutaraldehyde-polymerized bovine hemoglobin in plasma), HbRbr + Pls, (hemorrhage, transfused with glutaraldehyde-polymerized bovine hemoglobin in plasma), HbBSA (hemorrhage, transfused with the high-molecular weight fraction of a copolymer of bovine hemoglobin and bovine albumin, in plasma), Hb(ov)/PBS (hemorrhage, transfused with glutaraldehyde-polymerized ovine hemoglobin in PBS), HbRSA (hemorrhage, transfused with a copolymer of bovine hemoglobin and rat albumin), HbRbr/PBS (hemorrhage, transfused with a copolymer of bovine hemoglobin and rubrerythrin), Gf (hemorrhage, transfused with gelofusine), and pHb(b)/Gf (hemorrhage, transfused with glutaraldehyde-polymerized hemoglobin in gelofusine).

HBOCs were administered via intravenous route in a proportional volume with the blood effusion during hemorrhage *á jeun* under deep narcosis as previously described. Hemorrhagic status was induced under narcosis, by blood effusion from retroorbital plexus until the blood volume was at 30% of the TBV (total blood volume) of the rat. The retroorbital puncture method implies the use of a heparinized capillary glass tube in the retroorbital plexus, gently rotating the tube as it is advanced. Venous blood is collected in a 3.5 mL blood sampling tubes. The blood arterial tension (mmHg) of the animals was monitored every 10 minutes for the first two hours following administration of the HBOC and then every two hours for the next 8 hours and then again at 24 hours. Blood arterial tension monitoring was performed by Coda noninvasive blood pressure measurement system for rodents (Kent Scientific Corporation, Torrington, CT 06790, SUA). In the end, the animals were subject to deep isoflurane narcosis and blood was collected as previously described [25]. The acid-base status (pH, pO_2_, pCO_2_, cHCO_3_, BE(ecf), and BE(b)), ions such as Na^+^, K^+^, and Ca^2+^, glycaemia (Glu), total protein (TP) content, transferrin (Transf), lactate (Lac), oxidative stress (catalase, concentration of thiobarbituric acid reactive substances (TBARS)), renal function (creatinine, urea, uric acid, iron, and phosphate) were done according to our previous work. Immunological parameters (IgA, IgG and IgM, C3, and CRP) were assessed with an immunoturbidimetric method using anti-IgA, anti-IgG, and anti-IgM specific antiserum [32]. Complete blood counts were also performed as previously described for hemoglobin-based HBOC [25]. For iron histochemical analyses, liver, spleen and kidney were collected by animal dissection, fixed in neutral formalin solution (10%) and analyzed by the Perls staining method and routine H&E evaluation [25].

### 2.4. Statistical Analysis

Data are reported as the mean ± SEM unless otherwise indicated. The Gaussian distribution was checked by the Shapiro–Wilk normality test. One-way analysis of variance ANOVA, followed by Bonferroni's Multiple Comparison test procedure, was performed. Statistical significance was at *p* < 0.05 (95% confidence interval). Statistical values were obtained using GraphPad Prism version 5.0 for Windows (GraphPad Software, San Diego, California, USA).

## 3. Results and Discussion

**Figure 1:**
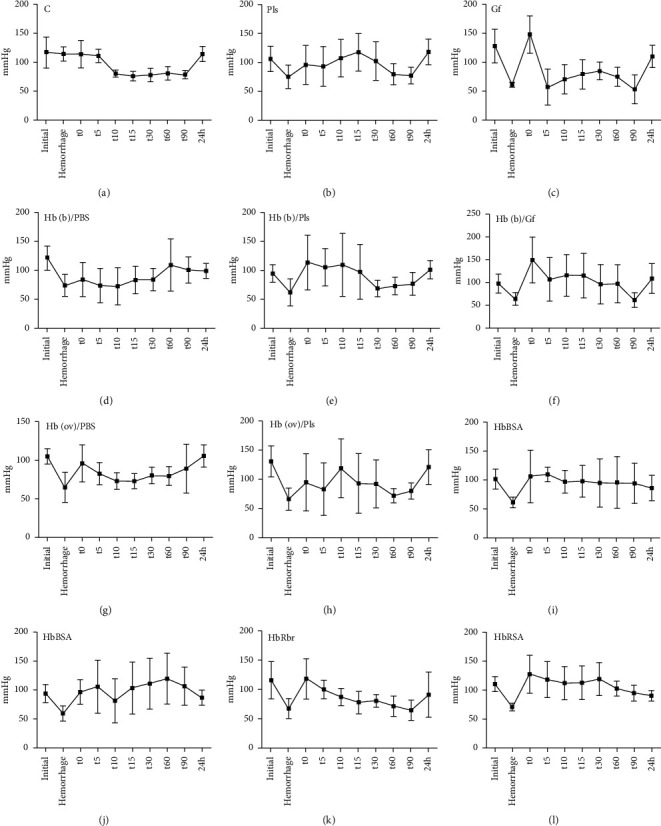
Evolution of blood arterial tension (mmHg) over the course of the experiment, measured before hemorrhage and then at given time points (0–90 minutes and 24 hours) after hemorrhage/treatment; see also supporting information Tables S1 and S2.

The arterial tension (AT) was monitored prior to experiments, then immediately after withdrawal of blood (∼2.5 mL, representing ∼30% of total blood volume related to animal weight, with the exception of the control group, C, for which only 0.5 mL was retrieved for use in biochemical assays), then for a further 90 minutes, and then again at 24 hours.  Table 1 shows survival rates for these experiments, while Figure 1 shows the evolution of AT values at the above-mentioned time points.

In all groups except the control and Hb(b)/PBS, AT is decreased to an average of 60–70 mmHg after hemorrhage. In the control group, one may note that even though hemorrhage was not performed, and instead a low amount of blood was retrieved for biochemical analyses, the animals did show a drop in AT (∼25 mmHg) at approximately the same time point at which the other groups did receive treatment. This suggests that part of the decrease in AT in all experimental groups is not only due to massive blood loss, but also due to the inherent stress induced by the experiment.

The AT changes induced by treatment range from −26 mmHg (only Hb(b)/PBS shows such further decrease, but this correlates to a smaller decrease induced by hemorrhage) to +31 mmHg for groups showing 100% survival rates and from +37 to +80 in groups with lower survival rates. It is generally known that hypertension is among the undesired side-effects of HBOC [33]; indeed, we find that here too increases of ∼40–80 mmHg immediately after transfusion correlate with poorer survival rates. By contrast, the difference between the final (at 24 hours) and initial AT no longer offers correlations with survival or major differences across groups.

Standardized plasma alone restored the survival rates from 75% (hemorrhage, no treatment) to 100%; the same was not true for gelofusine when administered alone, without an HBOC. These rates are accompanied by a slight drop in AT after treatment and a slight increase at the final time point is seen for Pls, with the opposite in Gf (the latter in line with previous observations [34]). One may conclude that, under these settings, gelofusine alone is not an efficient treatment for hemorrhage, despite the fact that its medical indications are precisely as a blood plasma replacement in cases of severe blood loss.

Serum albumin was previously proposed as a potentially useful ingredient in HBOCs, due to its protective redox effect *in vitro*, an effect which was later shown to some extent *in vivo* in top-up models [25]. In previous experiments, serum albumin when copolymerized with hemoglobin was shown to reduce the prooxidant reactivity of hemoglobin *in vitro* (as manifested in autoxidation rates as well as in the amounts of free radicals and of high-valent iron generated in reaction with a model oxidative stress species, hydrogen peroxide) as well as *in vivo* (as manifested in reduced amounts of met hemoglobin and of free radicals seen in top-up experiments with rats) [25]. Therefore, a sample of hemoglobin copolymerized with bovine serum albumin identical to the one employed successfully in previous top-up experiments (with 100% survival rate and with decreased oxidative stress markers in those previous experiments [25]) was employed in the present study. This sample, HbBSA, led to very poor survival rates in the hemorrhage experiments (25%). Taking into consideration that serum albumin is known for its immunogenic reactions, a second copolymer was prepared, where rat serum albumin (RSA) was employed instead of its bovine counterpart. For this sample, HbRSA, the survival rates were restored to 75%. These results suggest that if serum albumin is to be used in such experiments, its source must match the species on which transfusion is being performed.

The bacterial nonheme peroxidase, rubrerythrin, was previously shown to also prevent prooxidant reactivity in hemoglobin-rubrerythrin copolymers when compared to a simple Hb polymer prepared with glutaraldehyde. Despite the previous positive *in vitro* and *in vivo* top-up results [25, 27], the HbRbr copolymer led to very poor survival rates in the present experiments (50% when in PBS, 0%; when in Pls, not shown); these rates correlate with significantly increased AT values immediately after treatment and with immunological and histological analyses discussed below. Rbr must therefore be ruled out as a feasible ingredient in HBOC. The reason for this massive failure may either be in immunological reactions (unexpected since Rbr is a low-concentration ingredient, but confirmed by data shown below) or an excessive/uncontrolled peroxidase activity that leads to redox imbalance in the blood (in which case, much lower concentrations of Rbr would be required).

Figure 1 also shows the evolution of AT across the first 90 minutes after treatment with HBOC, following hemorrhage. The control group, following the milder handling incurred by extraction of blood for biochemical analyses but no larger-scale hemorrhage, shows a drop in AT by the first 10 minutes and then remains steady, never recovering to original values within the first 90 minutes. The vehicle controls, Pls and gelofusine, behave differently: while Pls mitigates the situation (and shows a slight increase over the course of the experiment), gelofusine leads to larger changes in AT, perhaps explaining the lack of improvement over the control group in terms of survival rates.

The Hb(b)/Pls group behaves relatively similar to Pls, with a notable difference that in some of the individuals AT values as low as 50 are recorded at times 10–30 min, with values as low as 60 maintained in some individuals even at 90 minutes; the onset of such low values was distinctly later in the Pls group and limited to less individuals. A further difference between the Pls and Hb(b)/Pls sample may be seen in the initial AT after treatment. While the average values are similar, the Hb(b)/Pls group includes individuals with values as high as 180 and 140 mmHg at this time point. Together, these differences may point to an increased risk of early hypertension and later hypotension induced by glutaraldehyde-polymerized bovine Hb. The Hb(b)/PBS group shows better AT values (as well as better survival results, cf.  Table 1) than Hb(b)/Pls; however, even here individuals with AT values as low as 30–50 mmHg and as high as 140–190 mmHg were seen. The Hb(b)/Gf group, while showing survival rates better than Hb(b)/Pls and equal to Hb(b)/PBS, still features extreme AT values ranging from below 50 to 190 mmHg (in line, notably, with similar trends seen in the Gf group), suggesting that of the three tested matrices, PBS is the safer one when used in conjunction with glutaraldehyde-polymerized bovine hemoglobin.

By contrast with bovine hemoglobin, ovine hemoglobin leads to milder average changes in AT over the first 90 minutes, either with Pls or with PBS as matrix. As in the case of the two bovine samples discussed above, PBS appears to afford less extreme AT values: 30–50 mmHg as well as 160–200 mmHg individual values are seen in the Hb(ov)/Pls group, while for Hb(ov)/PBS group the extreme values are 60 and 130 mmHg (with one additional value in one individual as high as 160, at *t* = 90 minutes). This implies that PBS is a safer matrix than plasma for these transfusion experiments with HBOC and that ovine Hb is a safer oxygen carrier than its bovine counterpart, a conclusion that mirrors the one drawn from the bovine Hb data discussed in the previous paragraph.

In the HbBSA group, the average AT in the surviving individuals tends to be higher than in other groups and there are strong variations across time, especially in the first 30 minutes; notably, there are also occasional extreme values as low as 30 mmHg and as high as 200 mmHg in members of this group. By contrast, the HbRSA group shows distinctly more stable AT values over time; this group also has no individual values below 80 mmHg and only one individual showing values higher than 140 mmHg (up to 170 mmHg). These data reinforce the conclusion that a serum albumin matching the receiving organism may be a reasonable choice as an ingredient in an HBOC. However, at least in the current experimental setting, HbRSA did not offer an improvement in survival rates over the control (untreated) group.

The HbRbr/PBS group shows one of the sharpest decreases in AT over the examined 90 minutes. This is due to several of the individuals displaying values as low as 30–50 mmHg at 90 minutes despite initial reasonable values. In one individual, the final value at 24 hours is still 50 mmHg, while three other individuals died before the 24-hour time point was reached. This points out severing hypotension as symptom induced by HbRbr/PBS.

**Figure 2:**
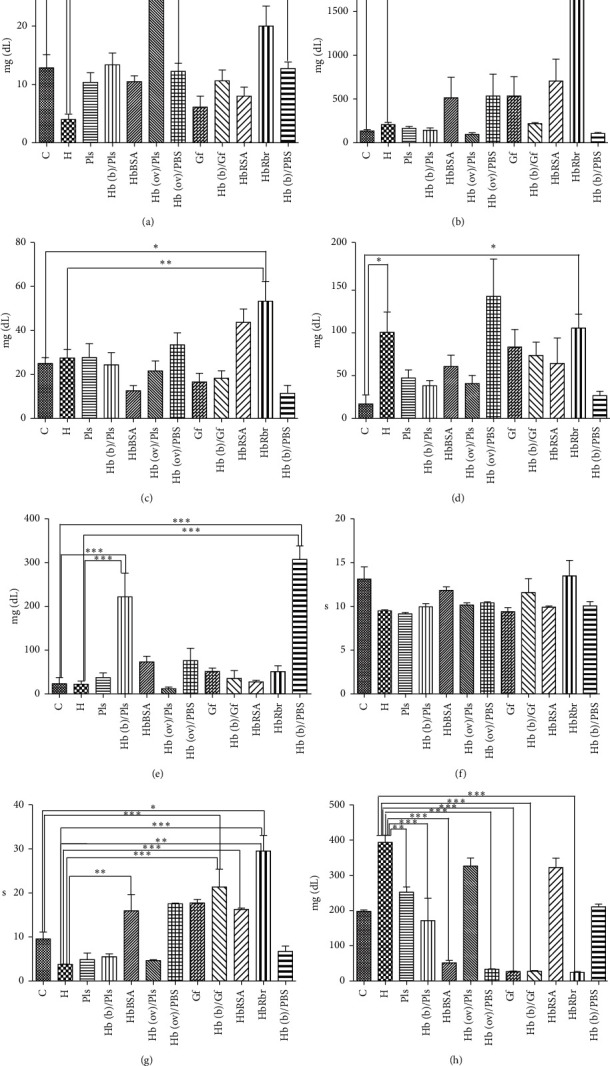
Immunological and clotting parameters of control and experimental groups. Values are expressed as mean ± SD. ^*∗*^Significant at *p* < 0.05; ^*∗∗*^significant at *p* < 0.01; ^*∗∗∗*^significant at *p* < 0.001^#^significant at *p* < 0.05; ^##^significant at *p* < 0.01; ^###^significant at *p* < 0.001 (compared with H). (a) IgA, (b) IgG, (c) IgM, (d) C3, (e) CRP, (f) PT, (g) aPTT, and (h) fibrinogen.

Immunological parameters (three immunoglobulins, alongside C-reactive protein and C3 complement protein) are shown in Figure 2. Hemorrhage alone induces a notable increase in IgG and in C3C, alongside a slight decrease in IgA with no changes in IgM and CRP. Treatment with the control vehicle, Pls or Gf, alleviates these changes; Pls also shows lower IgG than the H group, while Gf shows a distinctly higher level of IgG. The latter is not surprising, considering the amount of foreign protein (succinylated gelatin) in Gf. Pls also shows a slightly higher level of CRP than the H group.

HbRbr stands out with distinctly increased Ig and C3 levels and is thus immunologically incompatible for transfusion (though, in top-up models, with much smaller volumes of HBOC, no such response was noted) [25]. The two albumin groups on the other hand behave mostly similarly to each other, with IgG values increased only to levels similar to the Gf sample and hence unlikely to be of concern since Gf is a standard treatment in human medicine and with slight changes in CRP and C3C that are within the range already seen in the H group.

Glutaraldehyde-polymerized bovine Hb is currently approved for limited human use and may as such be taken as a reasonable reference point for a relatively acceptable HBOC material [25]. For the Hb(b)/Pls group, IgA, IgG, and IgM are very close to those of the control group, and in this respect they expectedly offer an improvement over the hemorrhage (H) as well as the Pls group; CRP shows a value intermediate between H and the control, while C3C is much higher. The other two samples of polymerized bovine Hb (pHb(b)/PBS, pHb(b)/Gf) show increases over pHb(b)/Pls either in IgG (pHb(b)/Gf) or in C3C (both) or in CRP (both) and IgG (pHb(b)/PBS). However, none of these changes are above the normal physiological limits and most of these differences do not carry statistical significance. Furthermore, polymerized ovine hemoglobin shows no notable increases with respect to its bovine counterparts: IgA and C3C are slightly increased, while IgG, IgG, and CRP are slightly decreased with respect to the control group and improved over the H group in terms of IgG and C3C; an exception is C3C for Hb(ov)/PBS (but not for Hb(ov)/Pls), which is clearly increased.

Figure 2 also shows relevant clotting parameters. The fibrinogen, an acute-phase protein, is doubled in the H group compared to the control, as expected after a trauma such as hemorrhage. Four samples display values intermediate between the H and the control groups, which is what one would expect of a reasonable HBOC candidate: Pls, Hb(ov)/Pls, Hb(b)/PBS, and HbRSA. In the rest of the samples, the fibrinogen levels are even below those of the control, slightly so for Hb(b)/Pls, but distinctly so for the rest. For these latter samples (Gf, Hb(b)/Gf, Hb(ov)/PBS, HbBSA, HbRSA, and HbRbr), clear increases are seen in aPTT (activated partial thromboplastin time, relevant for coagulation as well as for inflammation and immune response [35]) that mirror the decreases in fibrinogen. The latter observation suggests that a clotting imbalance may persist even at 24 hours after treatment with some HBOC candidates; it is nevertheless of note that none of the HBOC performs worse than the commercially available Gf (administered alone, with no HBOC) in this respect. Last but not least, Figure 2 also shows that, by contrast with fibrinogen and aPTT, the PT (prothrombin time) values show distinctly less variability among the sample.

Table 2 shows histological data collected from liver, lung, and kidney tissues. Hemorrhage is seen to have induced slight proliferation of Kupffer cells in liver and slight inflammation in lungs. Most of the HBOC candidates show worse diagnoses than the hemorrhage group. Exceptions are Hb(ov)/Pls for which only the slight inflammation in lung is seen and Hb(ov)/PBS for which the same inflammation is accompanied by (even smaller) Kupffer cell proliferation (liver) and lesions in renal tubuli. The Hb(b)/PBS and Hb(b)/Pls groups both show moderate dystrophies, to some extent also seen in Hb(b)/Gf; slight to moderate renal tubule lesion, slight liver necrosis, and a number of other irregularities are also notable in these three samples (with Hb(b)/Gf arguably faring better than the other two). The good performance of the ovine hemoglobin in these HBOC tests is in line with previous *in vitro* observations showing good resilience against oxidative and nitrosative stress agents [30].

In the BSA and RSA samples, the problems appear located at the liver and to some extent at the kidneys. The HbRbr groups show prominent histopathological changes, in line with the low survival rates and AT problems. In HbRbr/Pls (0% survival rate, not shown), strong hepatocyte dystrophies, liver stasis, and lung edema were noted. In HbRbr/PBS the histopathological changes were more moderate (granular degeneration of the hepatocytes, liver necrosis, and kidney tubular lesions with hydropic degeneration) compared to HbRbr/Pls in all three types of tissues.

As shown in Supporting Information Table S3, the hematocrit and hemoglobin levels are not affected in any statistically significant manner in any of the samples, except for an increase in Hb(b)/Pls and a decrease in Hb(o)/PBS. These changes are difficult to rationalize since the same proteins show no statistically significant changes compared to the control or to the hemorrhage group when the matrices are switched (Hb(b)/PBS and Hb(o)/Pls). Indeed, Hb(b) in other matrices than plasma is not known to increase hematocrit [36], nor is it doing so in the present set of experiments when dissolved in PBS and nor does Pls alone lead to increases in Hct. Overall, together with the AT and the survival data discussed above, PBS appears as a better matrix for bovine Hb-based HBOCs. For Hb(o)/PBS, the lower Hct and Hgb levels are also paralleled by a decrease in urea compared to the other HBOC (Table S5), with no other biochemical parameters altered and no correspondent finding in the histological analysis for the kidney.

As shown in Table S4 (Supporting Information), the pH of the blood is affected in a statistically significant manner only in the HbBSA sample (with accompanying changes in BE, suggesting a notable acid-base imbalance). Lower imbalances are seen in some of the other samples, suggesting that they have less to do with BSA *per se* than with Hb or with physiological imbalances elsewhere in the organism (see also below). Also, the renal function parameters, creatinine and urea, show statistically significant increases for the HbRbr sample (cf. Supporting Information Table S5 and in line with the histologically evidenced renal damage in Table 2). The oxidative stress markers (Table S6) do not vary significantly in most of the samples; exceptions are small decreases of the glutathione peroxidase in the Gf and HbRSA samples; it is unclear if these decreases are due to a decrease in oxidative stress or a depletion of GPX (glutathione peroxidase) due to increased oxidative stress.

Table 3 shows total protein, transferrin, glucose, and lactate levels. Notable changes are seen in transferrin, which is increased in most of the samples (possibly as a reaction to the excess iron brought in the system by the HBOC) and decreased in HbRbr (possibly as a result of depletion of transferrin again by excess iron). There are also distinctly lower levels of glucose in HbBSA and in Gf, which may be linked with the acid-base imbalances in the context of acidosis. Lactate levels, while decreased in the H group, are slightly increased in all of the HBOC groups, with the highest value seen in Gf. Slight acidosis and decreased survival rates compared to other solutions (albumin) have been reported before with Gf in medical trials [37, 38].

As shown in Supporting Information (Table S7), the iron levels are lower in almost all groups (except for Pls, Gf, HbRbr/PBS, and pHb(b)/Gf) compared to the H group; the latter in turn shows expectedly lower levels than the control group. These small iron levels may suggest that at 24 hours the administered HBOC may have already been mostly eliminated from the blood. Also of note, Table S8 shows decreased iron deposits at the liver (perhaps expectedly) in the H group, with no further changes in most groups, except for distinct increases in Hb(b)/PBS and Hb/Rbr, as well as a further decrease in group Gf. Other ions (cf. Table S7) do not show statistically significant changes, except for potassium in the case of HbBSA, likely in connection with the acidosis problem discussed above.

## 4. Conclusions

Among a set of glutaraldehyde-polymerized hemoglobin-based oxygen carriers (HBOC) employed for treating hemorrhagic shock, a bovine Hb-based HBOC restored the survival rate to 100% (though the effect was also dependent on the matrix in which the HBOC was dissolved with PBS faring better than standardized plasma or than gelofusine), which is in line with the previous data (though the gelofusine and plasma observations in relation with HBOC are to our knowledge novel). The analogous HBOC produced with ovine Hb (tested here for the first time, to our knowledge, in hemorrhagic shock settings) performs better than the ovine one, not only in terms of survival rates but also in histopathological and immunological analyses. This suggests that ovine hemoglobin would be the best raw material for HBOC development (as opposed to bovine Hb previously used or to human Hb whose supply is at the moment limited, unless recombinant protocols are shown to be operational at large scales). Copolymerization of hemoglobin with serum albumin, previously advocated as a strategy for preventing oxidative stress (where albumin would serve to absorb oxidizing equivalents), is now found to only yield reasonable results when employing albumin from the same species. When used as co-component in HBOC, rubrerythrin (an efficient peroxidase, also previously advocated as a strategy against oxidative stress side reactions in HBOC) leads to drastic drops in survival rates and to general damage at several physiological parameters, with severe immunological reactions, and must thus be discarded from the list of useful HBOC ingredients.

## Figures and Tables

**Table 1:** Survival rates at 24 hours.

Group	Survival (%)
C	100
H	75
Pls	100
Gf	75
Hb(b)/PBS	100
Hb(b)/Pls	75
Hb(b)/Gf	75
Hb(ov)/PBS	100
Hb(ov)/Pls	100
HbBSA	25
HbRSA	75
HbRbr/PBS	50

**Table 2:** Summary of histological findings for transfusion experiments in the present study. The symbols are H: hemorrhage, P: plasma, GVD: granular and vacuolar degeneration, DG: degeneration, VD/GD: vacuolar degeneration/granular degeneration, N: necrosis, S: stasis, K: Kupffer cells, I: inflammation, ED: edema, TL: tubular lesions, M: mesangial proliferation, USD: urinary space dilation, TD: tubular dilation; +/−: slight/absent changes, +: slight changes, ++: moderate changes, and +++: prominent changes.

Group	Liver	Lungs	Kidneys
C	*None*	*None*	*None*
H	+K	+I	+TD
Pls	VD++DG	+/−I	+/− M
Gf	+GVD	+/−I	+/−TL, +/− M
Hb(b)/PBS	VD+++DG, +N	+I	+TL, +/−M
Hb(b)/Pls	+++GVD, +N, +++K	+I	+++TL, +M, +++USD
Hb(b)/Gf	++GVD	+I	+TL, +USD
Hb(ov)/PBS	+/− K	+I	+/− TL
Hb(ov)/Pls	*None*	+I	*None*
HbBSA	+++GVD, ++N	+I	GD + DG, +TL
HbRSA	VD+++DG	+I	+TL, ++M
HbRbrPBS	GD + DG, +++N, +++K	+I	VD + DG, +++TL

**Table 3:** Transferrin, total proteins, glucose, and lactate in control and experimental animals.

Param.	C	H	Pls	Gf	Hb(b)/PBS	Hb(b)/Pls	Hb(b)/Gf	Hb(ov)/Pls	Hb(ov)/PBS	HbBSA	HbRSA	HbRbr
TP (g/dL)	10.19 ± 0.44	8.07 ± 0.10^*∗*^	8.65 ± 0.19	8.20 ± 0.65	9.10 ± 0.27	9.47 ± 0.21	10.08 ± 0.95^#^	9.20 ± 0.20	7.70 ± 0.46^*∗∗∗*^	9.91 ± 0.08	8.22 ± 0.14	8.05 ± 0.22^*∗*^
Transf (mg/dL)	14.25 ± 0.86	37.88 ± 2.66^*∗∗*^	18.75 ± 2.60	18.63 ± 4.60	17.13 ± 2.58^#^	36.13 ± 5.22^#^	16.13 ± 3.61^#^	26.13 ± 5.22	27.50 ± 6.13	19.00 ± 4.65	20.50 ± 6.13	9.87 ± 2.19^###^
Glu (mg/dL)	243.1 ± 17.98	259.8 ± 11.11	303.4 ± 12.72	162.5 ± 4.59^*∗,##*^	249.1 ± 7.39	230.1 ± 17.81	187.5 ± 27.24	256.5 ± 9.81	223.63 ± 0.89^###^	165.3 ± 22.38^*∗##*^		
Lac (mmol/L)	1.60 ± 0.09	1.38 ± 0.11	1.95 ± 0.51	3.21 ± 0.71^*∗,##*^	1.88 ± 0.15	2.05 ± 0.13	2.06 ± 0.24	1.85 ± 0.09	1.91 ± 0.30	1.78 ± 0.07		

^*∗*^Significant at *p* < 0.05; ^*∗∗*^significant at *p* < 0.01; ^*∗∗∗*^significant at *p* < 0.001. ^#^significant at *p* < 0.05; ^##^significant at *p* < 0.01; ^###^significant at *p* < 0.001 (compared with H). Values are expressed as mean ± SEM.

## Data Availability

Raw data of the study are available from authors upon request.

## Supplementary Materials

Supplementary Materials Table S1: blood arterial tension (mmHg) across the first 0–90 minutes after treatment with HBOC, following hemorrhage. Table S2: blood arterial tension (mmHg), and survival rates at 24 hours. Table S3: hematocrit and hemoglobin values of control and experimental groups. Values are expressed as mean ± SEM. Table S4: acid-base equilibrium of control and experimental animals. Values are expressed as mean ± SEM. Table S5: renal function parameters of control and experimental animals. Values are expressed as mean ± SEM. Table S6. Oxidative and nitrosative stress status of control and experimental animals, reflected by glutathione peroxidase and 3-nitrotyrosine. Values are expressed as mean ± SEM. Table S7: blood ion concentration of control and experimental animals. Values are expressed as mean ± SEM. Table S8. Iron deposits evaluation by Perls staining in liver. The evaluation of the histochemical reaction was done by Grover et al. (2015) method as follows: +/−, slight to absent reaction; +, slight reaction; ++, moderate reaction; +++, intense reaction.

## References

[1] Chang T. M. S. (2004). Hemoglobin-based red blood cell substitutes. *Artificial Organs*.

[2] Chang T. M. S. (2009). Nanobiotechnology for hemoglobin-based blood substitutes. *Critical Care Clinics*.

[3] Portoro I., Kocsis L., Herman P. (2008). Towards a novel haemoglobin-based oxygen carrier: euro-PEG-Hb, physico-chemical properties, vasoactivity and renal filtration. *Biochimica et Biophysica Acta (BBA)*.

[4] Sakai H., Tsuchida E. (2006). Performances of PEG-modified hemoglobin-vesicles as artificial oxygen carriers in microcirculation. *Clinical Hemorheology and Microcirculation*.

[5] Chen J.-Y., Scerbo M., Kramer G. (2009). A review of blood substitutes: examining the history, clinical trial results, and ethics of hemoglobin-based oxygen carriers. *Clinics*.

[6] Alayash A. I. (2000). Hemoglobin-based blood substitutes and the hazards of blood radicals. *Free Radical Research*.

[7] Scurtu V.-F., Moţ A., Silaghi-Dumitrescu R. (2013). Protein-based blood substitutes: recent Attempts at controlling pro-oxidant reactivity with and beyond hemoglobin. *Pharmaceuticals*.

[8] Alayash A. I. (2014). Blood substitutes: why haven’t we been more successful?. *Trends in Biotechnology*.

[9] Riess J. G. (2001). Oxygen carriers (“blood substitutes”)Raison d’Etre, chemistry, and some physiologyBlut ist ein ganz besondrer Saft1. *Chemical Reviews*.

[10] Winslow R. M. (2000). Blood substitutes: refocusing an elusive goal. Review. *British Journal of Haematology*.

[11] Tomita D., Kimura T., Hosaka H. (2013). Covalent core-shell architecture of hemoglobin and human serum albumin as an artificial O_2_ carrier. *Biomacromolecules*.

[12] Harris D. R., Palmer A. F. (2008). Modern cross-linking strategies for synthesizing acellular hemoglobin-based oxygen carriers. *Biotechnology Progress*.

[13] Deac F., Iacob B., Fischer-Fodor E., Damian G., Silaghi-Dumitrescu R. (2011). Derivatization of haemoglobin with periodate-generated reticulation agents: evaluation of oxidative reactivity for potential blood substitutes. *Journal of Biochemistry*.

[14] Scurtu F., Zolog O., Iacob B., Silaghi-Dumitrescu R. (2014). Hemoglobin-albumin cross-linking with disuccinimidyl suberate (DSS) and/or glutaraldehyde for blood substitutes. *Artificial Cells, Nanomedicine, and Biotechnology*.

[15] Fedorov A. N., Iarochkin V. S., Koziner V. B., Hachaturian A. A., Rozenberg G. I. (1978). Support of respiration and hemodynamics by complete blood replacement with artificial oxygen carriers: polyhemoglobinalbumin and polyhemoglobin. *Doklady Akademii Nauk SSSR*.

[16] Wong N. S. W., Chang T. M. S. (2007). Polyhemoglobin-fibrinogen: a novel oxygen carrier with platelet-like properties in a hemodiluted setting. *Artificial Cells, Blood Substitutes, and Biotechnology*.

[17] D’Agnillo F., Chang T. M. S. (1998). Polyhemoglobin-superoxide dismutase-catalase as a blood substitute with antioxidant properties. *Nature Biotechnology*.

[18] Chang T. M. S. (1997). *Blood Substitutes: Principles, Methods, Products and Clinical Trials*.

[19] Bian Y., Rong Z., Chang T. M. S. (2011). Polyhemoglobin-superoxide dismutase-catalase-carbonic anhydrase: a novel biotechnology-based blood substitute that transports both oxygen and carbon dioxide and also acts as an antioxidant. *Artificial Cells, Blood Substitutes, and Biotechnology*.

[20] Chang T. M. S. (1992). *Blood Substitutes and Oxygen Carriers*.

[21] Rudolph A. S., Philips W. T., Chang T. M. S. (1997). *Blood Substitutes: Principles, Methods, Products and Clinical Trials*.

[22] Tsuchida E., Sakai H., Horinouchi H., Kobayashi K. (2006). Hemoglobin-vesicles as a transfusion alternative. *Artificial Cells, Blood Substitutes, and Biotechnology*.

[23] Arkosi M., Scurtu F., Vulpoi A., Silaghi-Dumitrescu R., Kurtz D. (2017). Copolymerization of recombinant Phascolopsis gouldii hemerythrin with human serum albumin for use in blood substitutes. *Artificial Cells, Nanomedicine, and Biotechnology*.

[24] Toma V. A. A., Farcas A. D. D., Roman I. (2018). In vivo evaluation of hemerythrin-based oxygen carriers: Similarities with hemoglobin-based counterparts. *International Journal of Biological Macromolecules*.

[25] Toma V. A., Farcas A. D., Roman I. (2016). Comparative in vivo effects of hemoglobin-based oxygen carriers (HBOC) with varying prooxidant and physiological reactivity. *PLoS One*.

[26] Iacob B., Deac F., Cioloboc D., Damian G., Silaghi-Dumitrescu R. (2011). Hemoglobin-albumin crosslinked copolymers: reduced prooxidant reactivity. *Artificial Cells, Blood Substitutes, and Biotechnology*.

[27] Hathazi D., Mot A. C., Vaida A. (2014). Oxidative protection of haemoglobin and hemerythrin by cross-linking with a nonheme iron peroxidase: potentially improved oxygen carriers for use in blood substitutes. *Biomacromolecules*.

[28] Zolog O., Mot A., Deac F., Roman A., Fischer-Fodor E., Silaghi-Dumitrescu R. (2011). A new polyethyleneglycol-derivatized hemoglobin derivative with decreased oxygen affinity and limited toxicity. *The Protein Journal*.

[29] Fischer-Fodor E., Mot A., Deac F., Arkosi M., Silaghi-Dumitrescu R. (2011). Towards hemerythrin-based blood substitutes: comparative performance to hemoglobin on human leukocytes and umbilical vein endothelial cells. *Journal of Biosciences*.

[30] Hathazi D., Scurtu F., Bischin C. (2018). The reaction of oxy haemoglobin with nitrite: mechanism, antioxidant-modulated effect, and implications for blood substitute evaluation. *Molecules*.

[31] Deac F. V., Bolfa A. M., Magdas C., Sevastre B., Turc S., Silaghi-Dumitrescu R. (2010). Haemoglobin-based blood substitutes: which hemoglobin to use. *Romanian Journal of Biochemistry*.

[32] Toma V. A., Tigu A. B., Farcaș A. D. (2019). New aspects towards a molecular understanding of the allicin immunostimulatory mechanism via Colec12, MARCO, and SCARB1 receptors. *International Journal of Molecular Sciences*.

[33] Dunne J., Caron A., Menu P. (2006). Ascorbate removes key precursors to oxidative damage by cell-free haemoglobin in vitro and in vivo. *Biochemical Journal*.

[34] Strengers P. F. W., van Twuijver E. (2009). Blood, blood components, plasma, and plasma products. *Side Effects of Drugs Annual*.

[35] Long A. T., Kenne E., Jung R., Fuchs T. A., Renné T. (2016). Contact system revisited: an interface between inflammation, coagulation, and innate immunity. *Journal of Thrombosis and Haemostasis*.

[36] Jahr J. S., Mackenzie C., Pearce L. B., Pitman A., Greenburg A. G. (2008). HBOC-201 as an alternative to blood transfusion: efficacy and safety evaluation in a multicenter phase III trial in elective orthopedic surgery. *The Journal of Trauma: Injury, Infection, and Critical Care*.

[37] Spoelstra-de Ma A. M. E., Smorenberg A., Groeneveld A. B. J. (2017). Different effects of fluid loading with saline, gelatine, hydroxyethyl starch or albumin solutions on acid-base status in the critically ill. *PLoS One*.

[38] Akech S., Gwer S., Idro R. (2006). Correction: volume expansion with albumin compared to gelofusine in children with severe malaria: results of a controlled trial. *PLoS Clinical Trials*.

