# Supplementary material for: Glutaraldehyde-Polymerized Hemoglobin: In Search of Improved Performance as Oxygen Carrier in Hemorrhage Models
Source: Bioinorg Chem Appl. 2020 Sep 1;2020:1096573. doi: 10.1155/2020/1096573 (PMC7482000; doi:10.1155/2020/1096573)
Supplement: Supplementary Materials — Table S1: blood arterial tension (mmHg) across the first 0–90 minutes after treatment with HBOC, following hemorrhage. Table S2: blood arterial tension (mmHg), and survival rates at 24 hours. Table S3: hematocrit and hemoglobin values of control and experimental groups. Values are expressed as mean ± SEM. Table S4: acid-base equilibrium of control and experimental animals. Values are expressed as mean ± SEM. Table S5: renal function parameters of control and experimental animals. Values are expressed as mean ± SEM. Table S6. Oxidative and nitrosative stress status of control and experimental animals, reflected by glutathione peroxidase and 3-nitrotyrosine. Values are expressed as mean ± SEM. Table S7: blood ion concentration of control and experimental animals. Values are expressed as mean ± SEM. Table S8. Iron deposits evaluation by Perls staining in liver. The evaluation of the histochemical reaction was done by Grover et al. (2015) method as follows: +/−, slight to absent reaction; +, slight reaction; ++, moderate reaction; +++, intense reaction. [file 1096573.f1.docx]

Supporting Information

**Glutaraldehyde-polymerized hemoglobin: in search of improved performance as oxygen carrier in hemorrhage models**

**Anca D. Farcas^1,2,3^**^,a^ **Vlad Al. Toma^1,2,3,^** ^a^ **Ioana Roman^1^**, **Bogdan Sevastre^4^**, **Florina Scurtu^2^**, **Radu Silaghi-Dumitrescu^2^***

*corresponding author, rsilaghi@chem.ubbcluj.ro

^a^authors with equal contributions

^1^Institute of Biological Research Cluj-Napoca 400113, branch of NIRDBS Bucharest, Romania

^2^Department of Chemistry and Chemical Engineering, Babeș-Bolyai University, Cluj-Napoca 400028, Romania

^3^National Institute for Research and Development of Isotopic and Molecular Technologies, 400293 Cluj-Napoca, Romania

^4^Department of Pathophysiology, University of Agricultural Sciences and Veterinary Medicine, Cluj-Napoca, 400372, Romania

**Table S1.** Blood arterial tension (mmHg) across the first 0-90 minutes after treatment with HBOC, following hemorrhage.

| **Group** | **0** | **5** | **10** | **15** | **30** | **60** | **90** | ***d(0-90)^a^*** |
| --- | --- | --- | --- | --- | --- | --- | --- | --- |
| **C** | NA | NA | 81 | NA | 79 | 82 | 79 | *36* |
| **Pls** | 96 | 93 | 107 | 117 | 102 | 80 | 77 | *19* |
| **Gf** | 148 | 58 | 71 | 80 | 85 | 76 | 54 | *94* |
| **Hb(b)/PBS** | 84 | 74 | 73 | 83 | 84 | 109 | 100 | *-16* |
| **Hb(b)/Pls** | 113 | 105 | 109 | 97 | 69 | 73 | 77 | *36* |
| **Hb(b)/Gf** | 150 | 107 | 116 | 115 | 96 | 97 | 61 | *89* |
| **Hb(ov)/PBS** | 95 | 82 | 72 | 72 | 79 | 79 | 88 | *8* |
| **Hb(ov)/Pls** | 95 | 83 | 119 | 93 | 92 | 72 | 80 | *15* |
| **HbBSA** | 97 | 106 | 82 | 104 | 112 | 120 | 107 | *-10* |
| **HbRSA** | 127 | 118 | 112 | 112 | 119 | 103 | 95 | *32* |
| **HbRbr/PBS** | 119 | 100 | 87 | 78 | 81 | 72 | 64 | *56* |

^a^difference between time immediately after treatment (0) and 90 minutes.

**Table S2.** Blood arterial tension (mmHg) and survival rates at 24 hours.

| **Group** | **Initial** | **shock** | **treatment** | **final** | **d(shock)^a^** | **d(treatment)^b^** | **d(final)^c^** | **survival (%)^d^** |
| --- | --- | --- | --- | --- | --- | --- | --- | --- |
| **C** | 107 | 115 | 81 | 115 | *8* | -34 | *8* | 100 |
| **H** | 101 | 65 | NA | 89 | *-36* | NA | *-12* | 75 |
| **Pls** | 106 | 75 | 96 | 118 | *-31* | 21 | *12* | 100 |
| **Gf** | 128 | 62 | 148 | 110 | *-66* | 86 | *-18* | 75 |
| **Hb(b)/PBS** | 135 | 113 | 84 | 99 | *-22* | -29 | *15* | 100 |
| **Hb(b)/Pls** | 94 | 62 | 113 | 101 | *-32* | 51 | *7* | 75 |
| **Hb(b)/Gf** | 98 | 64 | 150 | 109 | *-34* | 86 | *11* | 75 |
| **Hb(ov)/PBS** | 104 | 64 | 95 | 105 | *-40* | 31 | *1* | 100 |
| **Hb(ov)/Pls** | 131 | 66 | 95 | 121 | *-65* | 29 | *-10* | 100 |
| **HbBSA** | 95 | 60 | 97 | 87 | *-35* | 37 | *-8* | 25 |
| **HbRSA** | 110 | 71 | 127 | 90 | *-39* | 56 | *-20* | 75 |
| **HbRbr/PBS** | 116 | 67 | 119 | 92 | *-49* | 52 | *-24* | 50 |

^a^difference between time points immediately after hemorrhage and prior to hemorrhage

^b^immediate change in AT induced by administration of treatment

^c^difference between final time point (24 hours) and time point immediately after treatment

^d^survivors after administration of treatment (at 24 hours)

**Table S3.** Hematocrit and hemoglobin values of Control and experimental groups. Values are expressed as mean ± SEM.

| **Param.** | **C** | **H 30’** | **H 24h** | **Pls** | **Gf** | **Hb(b)/PBS** | **Hb(b)/Pls** | **Hb(b)/Gf** | **Hb(ov)/PBS** | **Hb(ov)/Pls** | **HbBSA** |
| --- | --- | --- | --- | --- | --- | --- | --- | --- | --- | --- | --- |
| Hct  (%) | 36.75±1.48 | 34.88±0.35 | 34.50±2.10 | 35.88±4.63 | 35.38±2.22 | 41.63±3.98 | 49.50±1.66^#,¥^ | 26.75±1.85 | 22.50±1.04* | 34.75±5.38 | 36.00±1.62 |
| Hgb  (g/dL) | 12.54±0.50 | 11.88±0.09 | 11.70±0.73 | 12.23±1.60 | 12.03±0.74 | 14.15±1.34 | 16.76±0.56^#,¥^ | 9.15±0.64 | 7.40±0.37* | 11.88±1.83 | 12.38±0.55 |

* Significant at *p* < 0.05; ** Significant at *p* < 0.01; *** Significant at *p* < 0.001

# Significant at *p* < 0.05; ## Significant at *p* < 0.01; ### Significant at *p* < 0.001 (compared with (H30’))

¥ Significant at *p* < 0.05; ¥¥ Significant at *p* < 0.01; ¥¥¥ Significant at *p* < 0.001 (compared with (H24h))

**Table S4.** Acid base equilibrium of control and experimental animals. Values are expressed as mean ± SEM.

| **Param.** | **C** | **H 30’** | **H 24h** | **Pls** | **Gf** | **Hb(b)/PBS** | **Hb(b)/Pls** | **Hb(b)/Gf** | **Hb(ov)/PBS** | **Hb(ov)/Pls** | **HbBSA** |
| --- | --- | --- | --- | --- | --- | --- | --- | --- | --- | --- | --- |
| pH | 7.33±0.01 | 7.40±0.01 | 7.37±0.01 | 7.38±0.03 | 7.380.04 | 7.32±0.00 | 7.31±0.01^#^ | 7.41±0.00 | 7.38±0.01 | 7.34±0.01 | 7.31±0.01^##^ |
| pCO_2_  _(mmHg)_ | 54.83±1.94 | 46.08±2.90 | 54.56±2.18 | 53.50±5.74 | 49.99±1.54 | 60.03±3.25 | 59.05±2.67 | 51.15±1.09 | 50.61±0.93 | 60.89±2.71^#^ | 53.88±3.48 |
| pO_2_  _(mmHg)_ | 36.70±3.72 | 33.09±2.30 | 35.50±1.79 | 35.69±1.73 | 49.49±3.83^#^ | 35.58±5.32 | 37.53±2.31 | 49.66±2.41^#^ | 34.78±1.87 | 38.86±4.41 | 36.63±2.38 |
| cHCO_3_  _(mmol/L)_ | 29.23±0.44 | 28.78±0.97 | 31.84±0.86 | 30.71±0.75 | 28.23±0.43 | 31.61±1.13 | 30.21±0.62 | 33.23±0.41^*#^ | 30.46±0.13 | 33.10±0.86^#^ | 27.03±1.04^¥¥^ |
| BE(ecf)(mmol/L) | 3.38±0.44 | 4.13±0.80 | 6.62±0.84 | 5.70±0.43 | 2.78±0.66 | 5.70±1.05 | 4.10±0.51 | 8.67±0.42^***,##^ | 5.46±0.19 | 7.42±0.77^**^ | 0.80±0.87^¥¥¥^ |
| BE(b)  (mmol/L) | 2.32±0.40 | 3.50±0.63 | 5.30±0.70^**^ | 4.33±0.51 | 2.08±0.65 | 3.83±0.68 | 2.25±0.40^¥¥^ | 7.65±0.37^***,###^ | 4.78±0.17 | 5.61±0.36^**^ | -0.05±0.62^###,¥¥¥^ |

* Significant at *p* < 0.05; ** Significant at *p* < 0.01; *** Significant at *p* < 0.001 (compared with Control)

# Significant at *p* < 0.05; ## Significant at *p* < 0.01; ### Significant at *p* < 0.001 (compared with (H30’))

¥ Significant at *p* < 0.05; ¥¥ Significant at *p* < 0.01; ¥¥¥ Significant at *p* < 0.001 (compared with (H24h))

**Table S5.** Renal function parameters of control and experimental animals. Values are expressed as mean ± SEM.

| **Param.** | **C** | **H** | **Pls** | **Gf** | **Hb(b)/PBS** | **Hb(b)/Pls** | **Hb(b)/Gf** | **Hb/ov/Pls** | **Hb/ov/PBS** | **HbBSA** | **HbRSA** | **HbRbr** |
| --- | --- | --- | --- | --- | --- | --- | --- | --- | --- | --- | --- | --- |
| Crea  (mg/dL) | 1.20±0.04 | 1.20±0.06 | 0.85±0.18 | 1.30±0.07 | 1.40±0.04 | 1.36±0.08 | 1.25±0.10 | 1.21±0.03 | 1.08±0.03 | 1.50±0.05 | 1.05±0.01 | 1.85±0.08  ^***,###^ |
| Urea(mg/dL) | 34.25±3.27 | 26.50±0.75 | 27.88±2.66 | 31.13±2.00 | 34.13±1.02 | 33.50±2.02 | 30.88±1.56 | 27.75±0.52 | 8.75±1.26  ^***^,^###^ | 44.50±5.33^#^ | 28.38±1.10 | 106.1±9.03  ^***,###^ |

* Significant at *p* < 0.05; ** Significant at *p* < 0.01; *** Significant at *p* < 0.001

# Significant at *p* < 0.05; ## Significant at *p* < 0.01; ### Significant at *p* < 0.001 (compared with H)

**Table S6.** Oxidative and nitrosative stress status of control and experimental animals, reflected by glutathione peroxidase and 3-nitrotyrosine. Values are expressed as mean ± SEM.

| **Param.** | **C** | **H** | **Pls** | **Gf** | **Hb(b)/PBS** | **Hb(b)/Pls** | **Hb(b)/Gf** | **Hb(ov)/Pls** | **Hb(ov)/PBS** | **HbBSA** | **HbRSA** | **HbRbr** |
| --- | --- | --- | --- | --- | --- | --- | --- | --- | --- | --- | --- | --- |
| GPX  (UI) | 21.93±0.0 | 21.94±0.0 | 21.93±0.0 | 17.80±0.0^***,###^ | 21.92±0.0 | 21.91±0.0^##^ | 17.80±0.0^***,###^ | 21.91±0.0^##^ | 21.90±0.0 | 21.90±0.0^*,###^ | 17.80±0.0  ^***, ###^ | 21.91±0.0  ^##^ |
| 3-NTyr  (pg/mL) | 0.203±0.0 | 0.173±0.0 | 0.153±0.0 | 0.158±0.01 | 0.160±0.01 | 0.140±0.0 | 0.155±0.01 | 0.165±0.01 | 0.170±0.01 | 0.137±0.01 | 0.150±0.01 | 0.140±0.01 |

* Significant at *p* < 0.05; ** Significant at *p* < 0.01; *** Significant at *p* < 0.001

# Significant at *p* < 0.05; ## Significant at *p* < 0.01; ### Significant at *p* < 0.001 (compared with H)

**Table S7.**Blood ion concentration of control and experimental animals. Values are expressed as mean ± SEM.

| **Param.** | **C** | **H** | **Pls** | **Gf** | **Hb(b)/PBS** | **Hb(b)/Pls** | **Hb(b)/Gf** | **Hb(o)/PBS** | **Hb(ov)/Pls** | **HbBSA** | **HbRSA** | **HbRbr** |
| --- | --- | --- | --- | --- | --- | --- | --- | --- | --- | --- | --- | --- |
| Iron  (µg/dL) | 165.6±19.99 | 107.8±12.64 | 159.4±17.68 | 112.5±21.27 | 72.50±7.74^**^ | 71.13±7.09^**^ | 111.3±14.51 | 72.75±2.34  ^***^ | 75.88±10.83^**^ | 57.25±10.16^***^ | 76.13±6.84  ^**^ | 161.8±24.12 |
| Sodium  (mmol/L) | 146.0±1.30 | 146.6±0.92 | 143.9±0.69 | 145.6±0.77 | 143.1±1.87 | 147.4±0.86 | 143.9±1.69 | 142.6±0.18 | 143.9±1.04 | 147.8±0.31 | N.D. | N.D. |
| Potassium  (mmol/L) | 5.06±0.08 | 6.45±0.54 | 5.98±0.69 | 5.26±0.12 | 5.25±0.24 | 4.93±0.24 | 5.55±0.35 | 4.86±0.02 | 5.20±0.23 | 4.60±0.11^#^ | N.D. | N.D. |
| Calcium  (mg/dL) | 1.42±0.01 | 1.40±0.01 | 1.37±0.02 | 1.45±0.01 | 1.33±0.05 | 1.41±0.01 | 1.41±0.03 | 1.39±0.01 | 1.27±0.06 | 1.40±0.01 | N.D. | N.D. |

* Significant at *p* < 0.05; ** Significant at *p* < 0.01; *** Significant at *p* < 0.001

# Significant at *p* < 0.05; ## Significant at *p* < 0.01; ### Significant at *p* < 0.001 (compared with H)

**Table S8. Iron deposits evaluation by Pearls staining in liver.** The evaluation of the histochemical reaction was done by Grover et al. (2015) method as follows: **+/-**, slight to absent reaction, **+,** slight reaction, **++**, moderate reaction, **+++**, intense reaction.

| **Group** | **Reaction** | **Group** | **Reaction** |
| --- | --- | --- | --- |
| **C** | **++** | **pHb(ov)/PBS** | **-/+** |
| **H** | **+** | **pHb(ov)/Pls** | **+** |
| **H+Pls** | **-/+** | **HbBSA+PBS** | **+** |
| **Gf** | **-** | **HbRSA** | **+/++** |
| **pHb(b)/Pls** | **-/+** | **HbRbr + PBS** | **++** |
| **pHb(b)/PBS** | **++/+++** | **HbRbr+Pls** | **++++** |
| **pHb(b)/Gf** | **+** |  |  |
